# Response of microbial community composition and function to land use in mining soils of Xikuang Mountain in Hunan

**DOI:** 10.1371/journal.pone.0299550

**Published:** 2024-05-14

**Authors:** Jiao Yue, Dongpeng Zhang, Miaomiao Cao, Yukui Li, Qianwen Liang, Fei Liu, YuQiang Dong

**Affiliations:** School of life Sciences, Huaibei Normal University, Huaibei, China; Shandong University, CHINA

## Abstract

Nine land types in the northern mining area (BKQ) (mining land, smelting land, living area), the old mining area (LKQ) (whole-ore heap, wasteland, grassland), and southern mining area (NKQ) (grassland, shrubs, farmland) of Xikuang Mountain were chosen to explore the composition and functions of soil bacterial communities under different habitats around mining areas. The composition and functions of soil bacterial communities were compared among the sampling sites using 16S rRNA high-throughput sequencing and metagenomic sequencing. α diversity analysis showed the soil bacterial diversity and abundance in the old mining area were significantly higher than those in the northern mining area. β diversity analysis demonstrated that the soil bacterial community composition was highly similar among different vegetation coverages in the southern mining area. Microbial community function analysis showed the annotated KEGG function pathways and eggNOG function composition were consistent between the grassland of the old mining area and the grassland of the southern mining area. This study uncovers the soil bacterial community composition and functions among different habitats in the mining areas of Xikuang Mountain and will underlie soil ecosystem restoration in different habitats under heavy metal pollution around the mining areas there.

## 1 Introduction

Xikuang or Tin Deposit Mountain, located near Lengshuijiang City of Hunan Province, China, is named "the capital of antimony" and is one of major antimony ores worldwide [1]. Since the first mining for antimony ore in 1897 [2], this mountain has a mining history up to 120 years. However, excessive exploitation in mining areas have caused soil heavy metal pollution and thereby threaten the growth of plants, animals and microbes in the periphery. In the same soil environment, microbes are more sensitive to metal pollution than animals or plants [3]. Various microbial parameters including soil microbial community composition and biomass are very sensitive to environmental changes, and are considered as key indices to evaluate soil environment quality [4, 5]. Thus, studying the microbial diversity in soils polluted by heavy metals is very critical [6].

As the soil habitats in the mining areas are complex and diverse, some environmental factors affect microbial community distributions among different soil habitats [7, 8]. Vegetation is one of the major regulators of soil microbial community composition and activity [9]. Yang et al. found vegetation reconstruction in copper ailing directionally altered microbial community composition [10]. Han et al. reported that vegetation type can affect the quantity, composition and catabolism of soil microbial communities [11]. Zarraonaindia et al. reported that both vegetation types and soil types affected soil microbial communities [12]. Gao et al. found soil environmental factors significantly affected the diversity of microbial community composition [13]. Xue et al. showed that land use type significantly affected the biomass, diversity and composition of soil microbes [14]. The different land use types resulted from different mining activities, and thus were affected by heavy metal pollution differently. Hence, the soil microbial community composition will be adjusted slightly to adapt to different soil habitats [15].

High-throughput sequencing has been applied to analyze microbial communities in seawater, soils, human hand surface, and human distal intestinal tract [16]. With 16S rRNA and 18S rRNA high-throughput sequencing, Gao et al. uncovered the composition and diversity of microbial communities in ailing soils [17]. Reportedly, the composition and diversity of microbial communities in antimony-polluted areas were related to environmental factors [18]. Nevertheless, analysis of soil microbial communities involves not only microbial biomass and diversity measurement, but also microbial distribution patterns and functions [19]. Unluckily, the soil bacterial community composition and functions in different habitats of the 3 mining areas (the northern mining area, the old mining area, and the southern mining area) of Xikuang Mountain are still unknown. This study aimed to 1) explore bacterial community composition using 16S rRNA sequencing; 2) analyze soil microbial community functions under different sites of the same eco-environment using metagenomic sequencing.

## 2 Materials and methods

### 2.1 Sampling

The experimental sites were located at the Xikuang Mountain, Lengshuijiang City, Hunan Province. Mining and smelting activities have resulted in the pollution of soil and water with Sb. The average annual rainfall and temperature are 1222 mm and 18.6°C, respectively. The northern mining area (BKQ), the old mining area (LKQ), and the southern mining area (NKQ) of Xikuang Mountain were chosen. At each mining area, 3 sampling sites were set, which led to totally 9 sampling sites (Table 1). At each sampling site, 6 soil samples were collected from the topsoil (0–5 cm). The total number of samples is 54. The samples were all put into aseptic sealing bags, and stored in a cooler (with ice bags). The samples were immediately taken back to the laboratory. All procedures were performed in aseptic conditions as much as possible. All samples were processed with 16S rRNA high-throughput sequencing. Samples LKQ6 and NKQ7 were also tested with metagenomics analysis. The described study complied with all relevant regulations and our non-invasive sampling method required no specific research permits.

**Table 1:** Details of sampling sites.

Sampling site	Latitude and longitude	Land use	Details
BKQ1	27°46’6.09"N;111°29’24.20"E	Mining land	0.01 km away from the mining plant
BKQ2	27°46’24.28"N;111°29’36.15"E	Smelting land	near the mining plant
BKQ3	27°46’40.73"N;111°29’58.23"E	Living area	4 km away from the smelting land
LKQ4	27°47’38.71"N;111°29’47.69"E	Whole-ore heap land	near the abandoned ore heap
LKQ5	27°47’19.41"N;111°29’53.41"E	Wasteland	near the smelting plant
LKQ6	27°47’28.97"N;111°30’4.55"E	Grassland	2.5 km from the smelting plant
NKQ7	27°44’18.13"N;111°27’56.00"E	Grassland	about 3 km from the mining area
NKQ8	27°44’2.23"N;111°27’47.63"E	Shrubs	0.5 km from the mining plant
NKQ9	27°44’17.43"N;111°27’42.51"E	Farmland	1 km from the smelting plant

### 2.2 16S amplicon sequencing

Total DNA extraction, PCR amplification, and Illumina HiSeq. DNA from each sample was extracted using MN NucleoSpin 96 Soil kits, and DNA quality was detected via agarose gel electrophoresis. The V3—V4 region of bacterial 16S rRNA genes were amplified via polymerase chain reaction (PCR) using forward primer 338F and reverse primer 806R. The PCR amplification system was 10 μL, including 50 ng ± 20% genomic DNA, 0.3 μL of each of forward and reverse primers (10 μM), KOD FX Neo Buffer 5 μL, dNTP (2 mM each) 2 μL, and KOD FX Neo 0.2 μL, which were diluted by ddH_2_O to 10 μL. The PCR conditions were: pre-denaturation at 95°C × 5 min; 20 cycles (95°C × 30 s, annealing at 50°C × 30 s, extention at 72°C × 40 s); extention at 72°C × 7 min and retension at 4°C. PCR products were purified on columns using an OMEGA DNA purification kit. After 1.8% agarose gel electrophoresis at 120 V for 40 min, the target fragments were recovered using a Monarch DNA gel extraction kit and sent to Beijing Biomarker Technologies Co., Ltd. for construction and sequencing on an Illumina HiSeq 2500 platform.

Sequencing data processing. With the original data, the reads of the samples were spliced on FLASH 1.2.11 [20] to form raw tags. Then the raw tags were filtered on Trimmomatic 0.33 [21], forming clean tags. Finally, chimeras were removed using UCHIME 8.1 [22] to form effective tags.

The tags were clustered on Usearch [23] at the 97% likelihood level, forming operational taxonomic units (OTUs). The raw OUTs were clustered for low-content screening (abundance < 0.005%), forming final OUTs. The OTUs were taxonomically annotated on basis of the taxonomic database Silva [24].

### 2.3 Metagenomics sequencing

After genomic DNA detection showed the samples were qualified, DNA was fragmented ultrasonically. Then the DNA fragments were purified, end-repaired, and 3’-end added with A, followed by sequencing connection. Next, a sequence library was created via PCR amplification, and its quality was tested. The qualified library was sequenced on Illumina.

The raw tags after sequencing were filtered on Trimmomatic to form clean reads. The clean reads were metagenomically assembled on MEGAHIT [25] to filter out the contig sequences shorter than 300 bp. The assembly results were evaluated on QUAST [26]. The coding region in the genome was identified on MetaGeneMark 3.26 [27]. Redundancy was removed on cd-hit 4.6.6 [28] at the similarity threshold of 95% and the coverage threshold of 90%. Finally, the functions were annotated using a general database and an exclusive database.

### 2.4 Statistical analysis

The α diversity index of the samples was calculated on Mothur 1.30, and a rarefaction curve was plotted. The β diversity index was calculated on QIIME. Results were analyzed in R v.4.0.3, by principal co-ordinates analysis (PCoA) and analysis of similarities (ANOSIM), which are based on unweighted unifrac. The homogeneity of variance was compared using the least significant difference method (LSD) in one-factor analysis of variance. In case of variance heterogeneity, the microbial α diversity index and bacterial community relative abundance were compared among different mining areas using Dunnett’s T3 test. PCoA curves were plotted in R v.4.0.3 and analyzed. With silva as the reference database, taxonomic annotations were done using a naive Bayesian classifier. Then the microbe names were updated with LPSN (http://lpsn.dsm.de/). The taxonomical composition and abundance of the communities were analyzed at the phylum and class levels. The protein sequences of non-redundant genes were matched with the protein sequences recorded in both databases KEGG and eggNOG using BLAST. Then the function annotations from KEGG and eggNOG were analyzed.

## 3 Results

### 3.1 Microbial α diversity of soil samples

The library coverage rates of the samples were all above 99% (Table 2), indicating this sequencing result is valuable for analysis. The ACE, Chao1, Simpson, and Shannon indices were used to evaluate species richness and diversity. According to Table 2, the diversity and richness of bacterial communities in LKQ samples were significantly higher than those in BKQ samples (P<0.05).

**Table 2:** Microbial α diversity index in soils of different mining areas.

Sample	ACE	Chao1	Simpson	Shannon	Coverage/%
BKQ	652.84±157.42a	654.37±170.13b	0.9880±0.0047b	7.51±0.54b	99.51±0.09a
LKQ	898.22±55.30a	926.46±69.84a	0.9948±0.0011a	8.50±0.28a	99.24±0.18b
NKQ	832.73±20.38a	846.76±28.43ab	0.9911±0.0004ab	8.19±0.07ab	99.45±0.03ab
ANOVE F	5.142	5.085	4.449	6.031	4.512

The different lowercases indicate significantly different in the same column indicate the α diversity index is significantly different among the mining areas (P<0.05).

One-factor analysis of variance (ANOVA) showed the α diversity index of soil microbes was different among mining areas. The ACE index was not significantly different among the three mining areas (P > 0.05). The Chao1, Simpson and Shannon indices were all significantly different between BKQ and LKQ (F = 6.57, 5.96, and 7.79, respectively; all P < 0.05). Hence, significant differences in the abundance and diversity of soil bacterial communities only exist between the northern mining area and the old mining area.

### 3.2 Beta diversity analysis of soil bacterial communities

Beta diversity is a comparative analytical measure of the microbial community composition among the different samples. In this study, β diversity was calculated using principal coordinates analysis (PCoA) and Analysis of similarities (ANOSIM) based on unweighted UniFrac metric.

**Fig 1:**
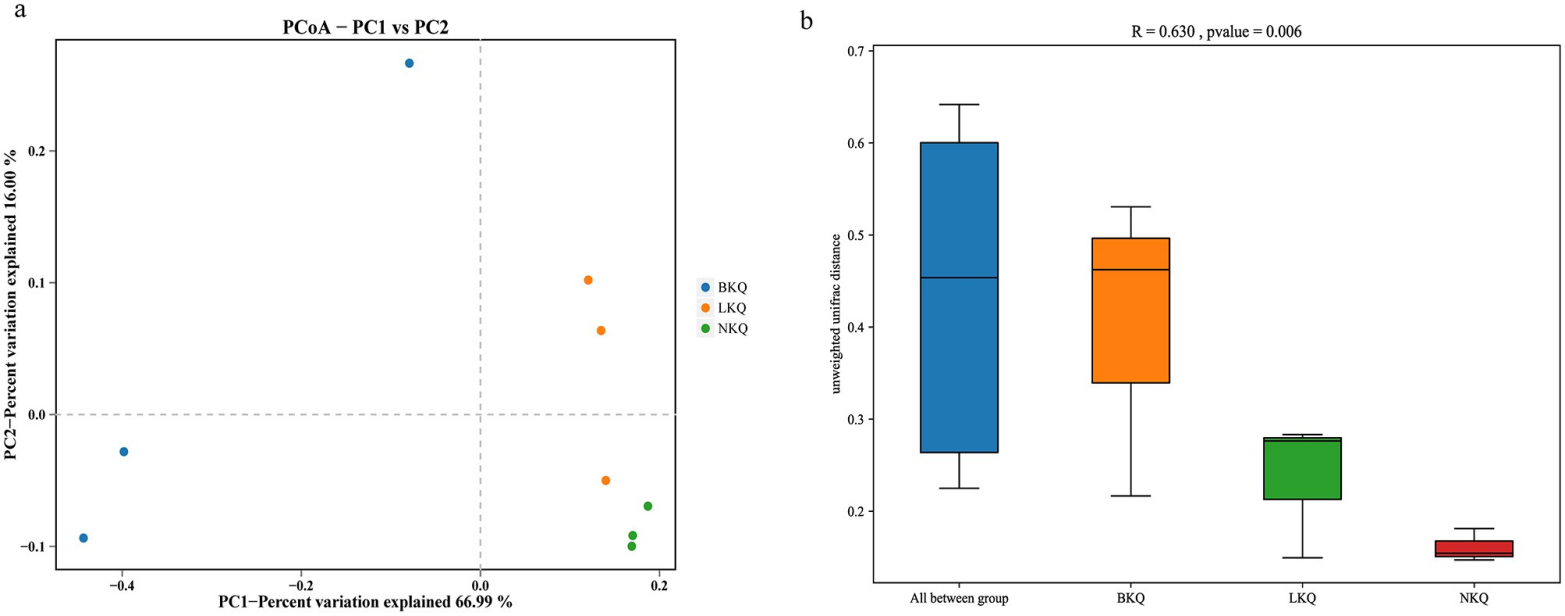
(a) PCoA and (b) ANOSIM based on unweighted UniFrac metric.

PCoA showed that the BKQ microbiome differed significantly from those of the LKQ and NKQ groups (Fig 1a). The results of ANOSIM indicated that inter-group differences were greater than intra-group differences, indicating that the soil bacterial community structure differed significantly among the different samples (R = 0.630, P = 0.006) (Fig 1b). PCoA plot and ANOSIM analysis together showed that the community composition of soil bacteria significantly differed among the different mining areas.

### 3.3 Analysis of soil bacterial community composition

**Fig 2:**
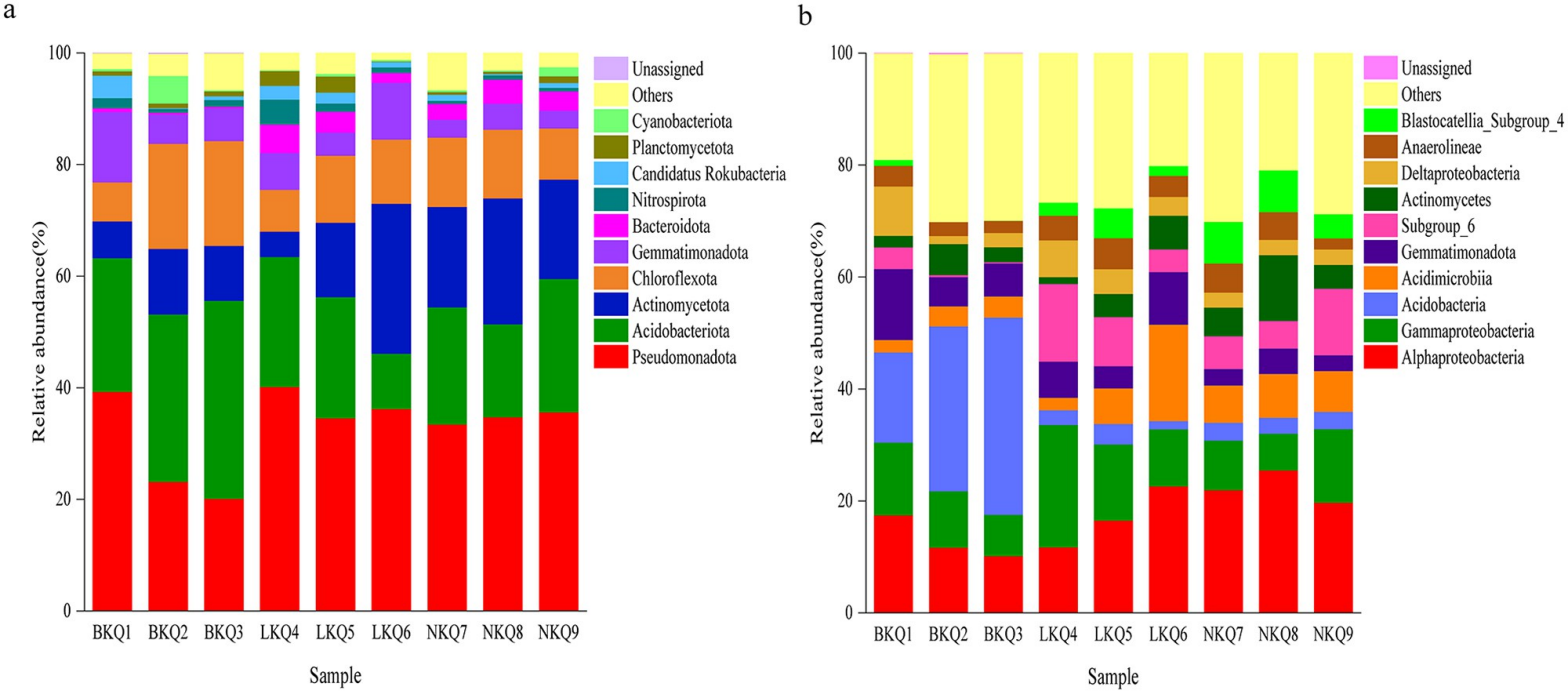
(a) Relative abundance of predominant bacterial at phyla and (b) class level.

The relative abundance of soil bacteria at the phylum level was shown in Fig 2a. The top 10 phyla ranked by relative abundance were Pseudomonadota (20.13%–40.15%), Acidobacteriota (9.91%–35.48%), Actinomycetota (4.54%–26.87%), Chloroflexota (6.97%–18.82%), Gemmatimonadota (3.16%–12.63%), Bacteroidota (0.28%–5.09%), Cyanobacteriota (0.14%–4.93%), Nitrospirota (0.59%–4.46%), Candidatus Rokubacteria (0.13%–4.00%), and Planctomycetota (0.13%–2.87%).

The top 10 bacterial phyla were sent to one-factor ANOVA to compare the differences in relative abundance of soil bacterial communities among different mining areas. Compared with the relative abundance of Bacteroidota (0.43% ± 0.22%) in BKQ, the relative abundance of Bacteroidota in LKQ (3.54% ± 1.66%) and NKQ (3.54% ± 0.74%) was significantly higher (F = 10.30, F = 48.44, both P < 0.05).

In the northern mining area, the predominant soil bacterial phyla were basically consistent among different habitats, but the relative abundance was significantly different. The top 4 dominant bacterial phyla in the soils of the mining land are Pseudomonadota (39.28%) > Acidobacteriota (23.97%) > Gemmatimonadota (12.63%) > Chloroflexota (6.97%). The top 4 dominant bacterial phyla in the soils of the smelting land are Acidobacteriota (30.01%) > Pseudomonadota (23.16%) > Chloroflexota (18.82%) > Actinomycetota (11.75%). The top 4 dominant bacterial phyla in the soils of the living area are Acidobacteriota (35.48%) > Pseudomonadota (20.13%)> Chloroflexota (18.79%) > Actinomycetota (9.83%). The relative abundance of Pseudomonadota, and Gemmatimonadota in the soils of mining land was far higher than in the other two habitats, and the relative abundance of Acidobacteriota and Chloroflexota in the soils of mining areas was far lower than in the two habitats.

In the old mining area, the predominant soil bacterial phyla were different among the sampling sites. The bacterial communities of whole-ore heap soils were dominated by Pseudomonadota and Acidobacteriota, and the sum of relative abundance was up to 63.46%. The bacterial communities of wasteland soils were dominated by Pseudomonadota, Acidobacteriota, Actinomycetota and Chloroflexota, and the sum of relative abundance was up to 81.63%. The bacterial communities of grassland soils were dominated by Pseudomonadota, Actinomycetota, Chloroflexota and Gemmatimonadota, and the sum of relative abundance was up to 84.73%. The relative abundance of Acidobacteriota in grassland soils was far lower than it in whole-ore heap soils, but the relative abundance of Actinomycetota was far higher than it in whole-ore heap or wasteland soils.

In the southern mining area, the predominant soil bacterial phyla were basically consistent among different habitats, and the difference in the relative abundance of the same phylum was low. The top 4 dominant bacterial phyla in the soils of the grasslands are Pseudomonadota (33.46%) > Acidobacteriota (20.99%) > Actinomycetota (17.99%) > Chloroflexota (12.43%). The top 4 dominant bacterial phyla in the soils of the shrubs are Pseudomonadota (34.77%) > Actinomycetota (22.55%) > Acidobacteriota (16.63%) > Chloroflexota (12.34%). The top 4 dominant bacterial phyla in the soils of the farmlands are Pseudomonadota (35.63%) > Acidobacteriota (23.89%) > Actinomycetota (17.80%) > Chloroflexota (9.16%). The top 4 dominant bacterial phyla were consistent among the soils of grassland, shrubs, and farmland in the southern mining area, including Pseudomonadota, Acidobacteriota, Actinomycetota, and Chloroflexota.

The relative abundance of soil bacteria at the class level was shown in Fig 2b. The top 10 bacterial classes ranked by relative abundance were Others(19.02% -30.13%), Alphaproteobacteria (10.18%–25.49%), Gammaproteobacteria (6.56%–21.85%), Acidobacteria (1.46%–35.20%), Acidimicrobiia (2.24%–17.21%), Gemmatimonadota (2.82%–12.62%), Actinomycetes (1.23%–11.75%), Deltaproteobacteria (1.42%–8.81%), Blastocatellia_Subgroup_4 (0.02%–7.48%), and Anaerolineae (1.99%–5.57%).

The top 10 bacterial classes were sent to one-factor ANOVA to compare the differences in pair relative abundance of soil bacterial communities among different mining areas. The relative abundance of Alphaproteobacteria (22.40% ± 2.91%), Acidimicrobiia (7.26% ± 0.59%) and Blastocatellia_Subgroup_4 (6.40% ± 1.83%) in the soils of NKQ was significantly higher than that of BKQ (F = 11.16, 46.97, and 29.61, respectively; all P < 0.05). The relative abundance of Blastocatellia_Subgroup_4 (6.40% ± 1.83%) in the soils of NKQ was significantly higher than compared with BKQ and LKQ (F = 29.61, F = 4.62, both P < 0.05).

In the northern mining area, the predominant soil bacterial classes were basically consistent among different habitats, but the relative abundance was different slightly. The top 4 dominant bacterial classes in the soils of the mining land are Alphaproteobacteria (17.45%) > Acidobacteria (16.07%) > Gammaproteobacteria (13.02%) > Gemmatimonadota (12.62%). The top 4 dominant bacterial classes in the soils of the smelting land are Acidobacteria (29.49%) > Alphaproteobacteria (11.68%) > Gammaproteobacteria (10.06%) > Gemmatimonadota (5.24%). The top 4 dominant bacterial classes in the soils of the living region are Acidobacteria (35.20%) > Alphaproteobacteria (10.18%) > Gammaproteobacteria (7.39%) > Gemmatimonadota (5.88%). The relative abundance of Alphaproteobacteria, Gammaproteobacteria and Gemmatimonadota in the soils of mining areas was far higher than in the other two habitats, and the relative abundance Acidobacteria and Others of in the soils of mining areas was far lower than in the two habitats.

In the old mining area, the predominant soil bacterial classes were basically consistent among different habitats, but the relative abundance was different. The top 4 dominant bacterial classes in the soils of the whole-ore heap are Gammaproteobacteria (21.85%) > Subgroup_6 (13.88%) > Alphaproteobacteria (11.74%) > Gemmatimonadota (6.43%). The top 4 dominant bacterial classes in the soils of the wasteland are Alphaproteobacteria (16.51%) > Gammaproteobacteria (13.62%) > Subgroup_6 (8.75%) > Acidimicrobiia (6.37%). The top 4 dominant bacterial classes in the soils of the grassland are Alphaproteobacteria (22.61%) > Acidimicrobiia (17.21%) > Gammaproteobacteria (10.23%) > Gemmatimonadota (9.36%). The relative abundance of Alphaproteobacteria, Acidimicrobiia in the soils of grassland was far higher than in the other two habitats, and the relative abundance Gammaproteobacteria in the soils of grassland was far lower than in the two habitats.

In the southern mining area, the predominant soil bacterial classes were different among the sampling sites. The soil bacterial communities of grassland were dominated by Alphaproteobacteria, as the relative abundance was up to 21.98%. The soil bacterial communities of shrubs were dominated by Alphaproteobacteria and Actinomycetes, as the sum of relative abundance was up to 37.24%. The soil bacterial communities of farmland were dominated by Alphaproteobacteria, Gammaproteobacteria and Subgroup_6, as the sum of relative abundance was up to 44.74%. The relative abundance of Actinomycetes in the soils of shrubs was far higher than in the other two habitats, and the relative abundance of Gammaproteobacteria, or Subgroup_6 of farmland soils was far higher than in the other two habitats. Relatively, the predominant soil bacterial classes were consistent among different habitats in the southern mining area.

### 3.4 Functions of microbial communities

**Fig 3:**
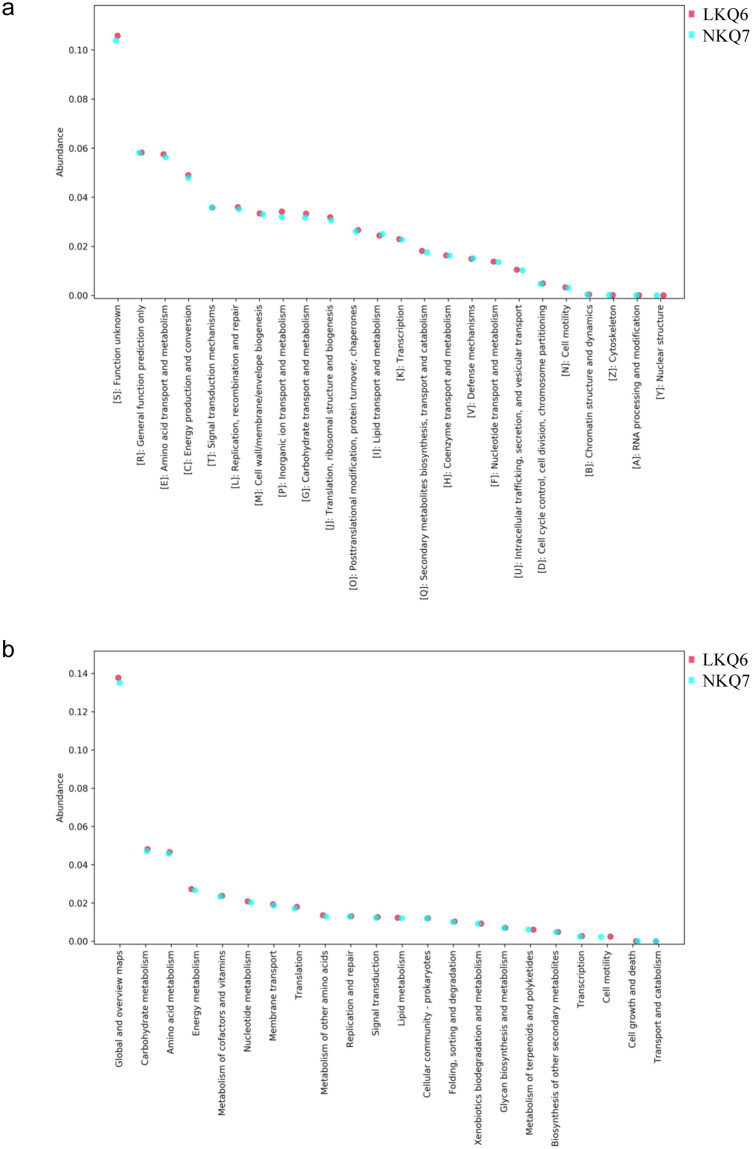
(a) Relative abundance matched with eggNOG database and (b) KEGG database.

The eggNOG functions of LKQ6 and NKQ7 were annotated (Fig 3a). The results indicate the compositions of the annotated functions are consistent between the 2 sampling sites, and the relative abundance is almost insignificantly different among the function clusters. It is suggested the compositions of annotated eggNOG function clusters are not significantly different between the grassland of the old mining area and the grassland of the southern mining area. The annotated eggNOG function cluster with the highest relative abundance is ’unknown’, followed by function prediction, amino acid transport and metabolism, and energy generation and conversion.

The KEGG functions of LKQ6 and NKQ7 were annotated (Fig 3b). The annotated KEGG function pathways from the 2 samples were very consistent, and nearly no significant difference in the relative abundance of any metabolic pathway was found. It is suggested the compositions of annotated KEGG function clusters are not significantly different between the grassland of the old mining area and the grassland of the southern mining area. The most annotated KEGG function type in the 2 sampling sites was ’global and review’, followed by carbohydrate metabolism, metabolism of amino acid, and energy metabolism.

The above results indicate the microbial community functions in the soils were consistent between the 2 sampling sites (grassland of the old mining area and grassland of the southern mining area).

## 4 Discussion

Soil bacterial communities are complex and diverse among different mining areas and different habitats, which shall be discussed by combining environment variables [15]. Guo et al. found that bacterial richness and diversity differed between sampling locations and ecological habitat had a significant effect on bacterial abundance [29], which is consistent with out findings. Chodak et al. thought heavy metal pollution very slightly affected the composition and diversity of soil microbial communities [30]. Yin et al. found the diversity and abundance of soil microbial communities decreased under long-term heavy metal pollution [31]. Narendrula-Kotha et al. found long-term contact with heavy metals for nearly 100 years reduced microbial abundance, but did not affect microbial diversity [32]. Heavy metal pollution reduced microbial abundance in sediments [33]. Random Forest (RF) model prediction from Sun et al. showed that the α diversity of microbial communities in Sb-polluted soils was an inverted U shape along with the variation of heavy metal pollution (Sb and As), as the α diversity slightly increased at low concentrations and rapidly dropped with the increasing pollution level [34]. After the pollution reached a certain level, the α diversity rose slowly with the aggravation of pollution [34].

The absolutely dominant bacterial phyla in the soils polluted by heavy metals around the antimony mining area were Pseudomonadota, Acidobacteriota, Actinomycetota, Chloroflexota, and Gemmatimonadota, and the sum of relative abundance reached 82.11%–94.64%. Wang et al. found Pseudomonadota, Acidobacteriota, Chloroflexota, Bacteroidota, Actinomycetota, Gemmatimonadota and Cyanobacteriota were predominance in the communities of Sb-polluted soils(Xikuangshan Sb mine area, Lengshuijiang, Hunan Province) [35]. Sun et al. found members of Pseudomonadota, Bacteroidota, Acidobacteriota and Actinomycetota were dominant in Sb/As-polluted soils [36]. These results are basically consistent with our study.

Kenarova et al. reported that the predominant bacteria in the soils of mining areas under heavy metal pollution may be tolerant against heavy metals to some extent [37]. Pseudomonadota has strong adaptability and is the most common bacterial phylum in nature [38]. Pseudomonadota can survive in extreme conditions and is considered to have potential to remediate heavy metal pollution [39]. Acidobacteriota is ubiquitous and is among the abundant bacterial phyla in soils [40]. Naether et al. thought Acidobacteriota accounted for 20% of all bacteria on average, and the diversity of Acidobacteriota differed among different ecoenvironments at the same site, and even the community composition of Acidobacteriota was significantly different among different sites of the same ecoenvironment [41]. Janssen et al. found the richest members of soil bacteria were from Pseudomonadota and Acidobacteriota [40]. Actinomycetota is widely distributed in different habitats, including terrestrial and aquatic environments, and is highly adaptive to adverse environments. Xiao et al. studied Sb-rich ailing and found Actinomycetota accounted for 10.3% of all bacteria [18]. Chloroflexota is involved in the biogeochemical cycling of carbon and nitrogen and can degrade toxic substances in soils [42, 43]. Gemmatimonadota is among the first nine phyla found in soils, accounting for 2% of soil bacterial communities, and is pivotal in terrestrial ecosystems [44]. The distribution of Gemmatimonadota in soils is dependent on the availability of moisture in soils, and cannot tolerate the moisture fluctuation due to dry and wet circulation. Moreover, the distribution of Gemmatimonadota is restricted by soils [45].

Moreover, the functional pathways of soil microbial communities were almost not different between LKQ6 and NKQ7. The above results indicate the functions of soil microbial communities may be consistent among different mining areas of the same habitat. Ezeokoli et al. found bacterial communities were affected by the differences in soil history and location, but the abundance of bacterial communities differed among different types of soils [46]. In comparison, the functions of bacterial communities were not significantly different, indicating the functions of bacterial communities are redundant among different soil types. These findings are consistent with our study.

The diversity and functions of soil bacterial communities among different habitats around the mining areas of Xikuang Mountain were analyzed, which will help with biological remediation of heavy-metal-polluted soils in the periphery of mining areas. However, we only preliminarily studied the functions of bacterial communities, so further research is needed to uncover the functions of bacterial communities, and even the existence and expressions of functional genes.

## 5 Conclusions

Generally, the soil bacterial communities of the 3 sampling sites were largely different. The soil bacterial community compositions varied significantly among different land use types in the northern mining area and the old mining area. In comparison, the soil bacterial community compositions did not vary significantly among different vegetation types in the southern mining area. The diversity and abundance of soil bacterial communities in LKQ are significantly higher than in BKQ (P<0.05). The relative abundance of some predominant bacterial phyla and classes are significantly different in the soils among different mining areas. The functions of bacterial communities are basically consistent between the grassland of the old mining area and the grassland of the southern mining area, indicating the functions of soil bacterial communities are redundant. The findings are expected to theoretically underlie ecosystem restoration in soils under heavy metal pollution around the mining areas.

## References

[1] He M. Distribution and phytoavailability of antimony at an antimony mining and smelting area, Hunan, China. Environ Geochem Health. 2007;29(3):209–19. doi: 10.1007/s10653-006-9066-9 17351815

[2] Fu Z, Wu F, Amarasiriwardena D, Mo C, Liu B, Zhu J, et al. Antimony, arsenic and mercury in the aquatic environment and fish in a large antimony mining area in Hunan, China. Sci Total Environ. 2010;408(16):3403–10. doi: 10.1016/j.scitotenv.2010.04.031 20452645

[3] Wang A, He M, Ouyang W, Lin C, Liu X. Effects of antimony (III/V) on microbial activities and bacterial community structure in soil. Sci Total Environ. 2021;789:148073. doi: 10.1016/j.scitotenv.2021.148073 34323828

[4] Liao M, Xie XM. Effect of heavy metals on substrate utilization pattern, biomass, and activity of microbial communities in a reclaimed mining wasteland of red soil area. Ecotoxicol Environ Saf. 2007;66(2):217–23. doi: 10.1016/j.ecoenv.2005.12.013 16488009

[5] Wang X, He M, Xi J, Lu X. Antimony distribution and mobility in rivers around the world’s largest antimony mine of Xikuangshan, Hunan Province, China. Microchemical Journal. 2011;97(1):4–11. doi: 10.1016/j.microc.2010.05.011

[6] Guo H, Nasir M, Lv J, Dai Y, Gao J. Understanding the variation of microbial community in heavy metals contaminated soil using high throughput sequencing. Ecotoxicol Environ Saf. 2017;144:300–6. doi: 10.1016/j.ecoenv.2017.06.048 28645031

[7] Vinhal-Freitas IC, Corrêa GF, Wendling B, Bobuľská L, Ferreira AS. Soil textural class plays a major role in evaluating the effects of land use on soil quality indicators. Ecological Indicators. 2017;74:182–90. doi: 10.1016/j.ecolind.2016.11.020

[8] Yin Y, Wang X, Hu Y, Li F, Cheng H. Soil bacterial community structure in the habitats with different levels of heavy metal pollution at an abandoned polymetallic mine. J Hazard Mater. 2023;442:130063. doi: 10.1016/j.jhazmat.2022.130063 36182879

[9] Wallenius K, Rita H, Mikkonen A, Lappi K, Lindström K, Hartikainen H, et al. Effects of land use on the level, variation and spatial structure of soil enzyme activities and bacterial communities. Soil Biology and Biochemistry. 2011;43(7):1464–73. doi: 10.1016/j.soilbio.2011.03.018

[10] Yang TT, Liu J, Chen WC, Chen X, Shu HY, Jia P, et al. Changes in microbial community composition following phytostabilization of an extremely acidic Cu mine tailings. Soil Biology and Biochemistry. 2017;114:52–8. doi: 10.1016/j.soilbio.2017.07.004

[11] Han XM, Wang RQ, Liu J, Wang MC, Zhou J, Guo WH. Effects of vegetation type on soil microbial community structure and catabolic diversity assessed by polyphasic methods in North China. J Environ Sci (China). 2007;19(10):1228–34. doi: 10.1016/s1001-0742(07)60200-9 18062422

[12] Zarraonaindia I, Owens SM, Weisenhorn P, West K, Hampton-Marcell J, Lax S, et al. The soil microbiome influences grapevine-associated microbiota. mBio. 2015;6(2). doi: 10.1128/mBio.02527-14 25805735 PMC4453523

[13] Gao TP, Fu JW, Zuo MB, Liu YB, Xu DH, Chang GH, et al. Study on the Rhizosphere Soil Microbial Community Structure Associated with Five Land Use Types in Jinchuan Mining Area. E3S Web of Conferences. 2021;237. doi: 10.1051/e3sconf/202123701010

[14] Xue P, Minasny B, McBratney AB. Land-use affects soil microbial co-occurrence networks and their putative functions. Applied Soil Ecology. 2022;169. doi: 10.1016/j.apsoil.2021.104184

[15] Zhao X, Huang J, Lu J, Sun Y. Study on the influence of soil microbial community on the long-term heavy metal pollution of different land use types and depth layers in mine. Ecotoxicol Environ Saf. 2019;170:218–26. doi: 10.1016/j.ecoenv.2018.11.136 30529916

[16] Zhang T, Shao MF, Ye L. 454 pyrosequencing reveals bacterial diversity of activated sludge from 14 sewage treatment plants. ISME J. 2012;6(6):1137–47. doi: 10.1038/ismej.2011.188 22170428 PMC3358032

[17] Gao T, Wang X, Liu Y, Wang H, Zuo M, He Y, et al. Characteristics and diversity of microbial communities in lead-zinc tailings under heavy metal stress in north-west China. Lett Appl Microbiol. 2022;74(2):277–87. doi: 10.1111/lam.13608 34822179

[18] Xiao E, Krumins V, Dong Y, Xiao T, Ning Z, Xiao Q, et al. Microbial diversity and community structure in an antimony-rich tailings dump. Appl Microbiol Biotechnol. 2016;100(17):7751–63. doi: 10.1007/s00253-016-7598-1 27188777

[19] Hill GT, Mitkowski NA, Aldrich-Wolfe L, Emele LR, Jurkonie DD, Ficke A, et al. Methods for assessing the composition and diversity of soil microbial communities. Applied Soil Ecology. 2000;15(1):25–36. doi: 10.1016/s0929-1393(00)00069-x

[20] Tanja M, Salzberg SL. FLASH: fast length adjustment of short reads to improve genome assemblies. Bioinformatics. 2011;(21):21. doi: 10.1093/bioinformatics/btr507 21903629 PMC3198573

[21] Bolger AM, Lohse M, Usadel B. Trimmomatic: a flexible trimmer for Illumina sequence data. Bioinformatics. 2014;30(15):2114–20. doi: 10.1093/bioinformatics/btu170 24695404 PMC4103590

[22] Edgar RC, Haas BJ, Clemente JC, Quince C, Knight R. UCHIME improves sensitivity and speed of chimera detection. Bioinformatics. 2011;27(16):2194. doi: 10.1093/bioinformatics/btr381 21700674 PMC3150044

[23] Edgar RC. UPARSE: highly accurate OTU sequences from microbial amplicon reads. Nat Methods. 2013;10(10):996–8. doi: 10.1038/nmeth.2604 23955772

[24] Quast C, Pruesse E, Yilmaz P, Gerken J, Glckner FO. The SILVA ribosomal RNA gene database project: Improved data processing and web-based tools. Nucleic Acids Research. 2012;41(D1). doi: 10.1093/nar/gks1219 23193283 PMC3531112

[25] Li D, Liu CM, Luo R, Sadakane K, Lam TW. MEGAHIT: an ultra-fast single-node solution for large and complex metagenomics assembly via succinct de Bruijn graph. Bioinformatics. 2015;31(10):1674–6. doi: 10.1093/bioinformatics/btv033 25609793

[26] Gurevich A, Saveliev V, Vyahhi N, Tesler G. QUAST: quality assessment tool for genome assemblies. Bioinformatics. 2013;29(8):1072–5. doi: 10.1093/bioinformatics/btt086 23422339 PMC3624806

[27] Tang S, Borodovsky M. Ab Initio Gene Identification in Metagenomic Sequences. Encyclopedia of Metagenomics. 2013. p. 1–8. doi: 10.1007/978-1-4614-6418-1_440-1 PMC2896542 20403810

[28] Fu L, Niu B, Zhu Z, Wu S, Li W. CD-HIT: accelerated for clustering the next-generation sequencing data. Bioinformatics. 2012;28(23):3150–2. doi: 10.1093/bioinformatics/bts565 23060610 PMC3516142

[29] Guo D, Fan Z, Lu S, Ma Y, Nie X, Tong F, et al. Changes in rhizosphere bacterial communities during remediation of heavy metal-accumulating plants around the Xikuangshan mine in southern China. Scientific Reports. 2019;9(1). doi: 10.1038/s41598-018-38360-2 30760787 PMC6374380

[30] Chodak M, Gołębiewski M, Morawska-Płoskonka J, Kuduk K, Niklińska M. Diversity of microorganisms from forest soils differently polluted with heavy metals. Applied Soil Ecology. 2013;64:7–14. doi: 10.1016/j.apsoil.2012.11.004

[31] Yin H, Niu J, Ren Y, Cong J, Zhang X, Fan F, et al. An integrated insight into the response of sedimentary microbial communities to heavy metal contamination. Sci Rep. 2015;5:14266. doi: 10.1038/srep14266 26391875 PMC4585741

[32] Narendrula-Kotha R, Nkongolo KK. Bacterial and fungal community structure and diversity in a mining region under long-term metal exposure revealed by metagenomics sequencing. Ecological Genetics and Genomics. 2017;2:13–24. doi: 10.1016/j.egg.2016.11.001

[33] Shen Z, Wang F, Liang Y, Li Y, Liu Q, Liu F. Diversity and functions of microbes in surface sediments under heavy metal pollution of western Chaohu Lake. Lett Appl Microbiol. 2022;75(5):1093–102. doi: 10.1111/lam.13627 34890483

[34] Sun X, Li B, Han F, Xiao E, Xiao T, Sun W. Impacts of Arsenic and Antimony Co-Contamination on Sedimentary Microbial Communities in Rivers with Different Pollution Gradients. Microb Ecol. 2019;78(3):589–602. doi: 10.1007/s00248-019-01327-5 30725170

[35] Wang N, Zhang S, He M. Bacterial community profile of contaminated soils in a typical antimony mining site. Environ Sci Pollut Res Int. 2018;25(1):141–52. doi: 10.1007/s11356-016-8159-y 28039624

[36] Sun X, Li B, Han F, Xiao E, Wang Q, Xiao T, et al. Vegetation type impacts microbial interaction with antimony contaminants in a mining-contaminated soil environment. Environ Pollut. 2019;252(Pt B):1872–81. doi: 10.1016/j.envpol.2019.06.070 31374407

[37] Kenarova A, Radeva G, Traykov I, Boteva S. Community level physiological profiles of bacterial communities inhabiting uranium mining impacted sites. Ecotoxicology and Environmental Safety. 2014;100:226–32. doi: 10.1016/j.ecoenv.2013.11.012 24315773

[38] Spain AM, Krumholz LR, Elshahed MS. Abundance, composition, diversity and novelty of soil Proteobacteria. ISME J. 2009;3(8):992–1000. doi: 10.1038/ismej.2009.43 19404326

[39] Karelová E, Harichová J, Stojnev T, Pangallo D, Ferianc P. The isolation of heavy-metal resistant culturable bacteria and resistance determinants from a heavy-metal-contaminated site. Biologia. 2010;66(1):18–26. doi: 10.2478/s11756-010-0145-0

[40] Janssen PH. Identifying the dominant soil bacterial taxa in libraries of 16S rRNA and 16S rRNA genes. Appl Environ Microbiol. 2006;72(3):1719–28. doi: 10.1128/AEM.72.3.1719-1728.2006 16517615 PMC1393246

[41] Naether A, Foesel BU, Naegele V, Wust PK, Weinert J, Bonkowski M, et al. Environmental factors affect Acidobacterial communities below the subgroup level in grassland and forest soils. Appl Environ Microbiol. 2012;78(20):7398–406. doi: 10.1128/AEM.01325-12 22885760 PMC3457104

[42] Zhang B, Kong W, Wu N, Zhang Y. Bacterial diversity and community along the succession of biological soil crusts in the Gurbantunggut Desert, Northern China. J Basic Microbiol. 2016;56(6):670–9. doi: 10.1002/jobm.201500751 26947139

[43] Milobedzka A, Witeska A, Muszynski A. Factors affecting population of filamentous bacteria in wastewater treatment plants with nutrients removal. Water Sci Technol. 2016;73(4):790–7. doi: 10.2166/wst.2015.541 26901721

[44] DeBruyn JM, Nixon LT, Fawaz MN, Johnson AM, Radosevich M. Global biogeography and quantitative seasonal dynamics of Gemmatimonadetes in soil. Appl Environ Microbiol. 2011;77(17):6295–300. doi: 10.1128/AEM.05005-11 21764958 PMC3165389

[45] Liu Z, Zhou H, Xie W, Yang Z, Lv Q. Long-term effects of maize straw return and manure on the microbial community in cinnamon soil in Northern China using 16S rRNA sequencing. PLoS One. 2021;16(4):e0249884. doi: 10.1371/journal.pone.0249884 33886593 PMC8062091

[46] Ezeokoli OT, Bezuidenhout CC, Maboeta MS, Khasa DP, Adeleke RA. Structural and functional differentiation of bacterial communities in post-coal mining reclamation soils of South Africa: bioindicators of soil ecosystem restoration. Sci Rep. 2020;10(1):1759. doi: 10.1038/s41598-020-58576-5 32019965 PMC7000389

